# Activation of the MAPK network provides a survival advantage during the course of COVID-19-induced sepsis: a real-world evidence analysis of a multicenter COVID-19 Sepsis Cohort

**DOI:** 10.1007/s15010-024-02325-7

**Published:** 2024-06-19

**Authors:** Andrea Witowski, Lars Palmowski, Tim Rahmel, Hartmuth Nowak, Stefan F. Ehrentraut, Christian Putensen, Thilo von Groote, Alexander Zarbock, Nina Babel, Moritz Anft, Barbara Sitek, Thilo Bracht, Malte Bayer, Maike Weber, Christina Weisheit, Stephanie Pfänder, Martin Eisenacher, Michael Adamzik, Rump Katharina, Björn Koos, Dominik Ziehe, the CovidDataNet.NRW research group, Andrea Witowski, Lars Palmowski, Tim Rahmel, Hartmuth Nowak, Christian Putensen, Thilo von Groote, Alexander Zarbock, Nina Babel, Moritz Anft, Barbara Sitek, Thilo Bracht, Malte Bayer, Maike Weber, Christina Weisheit, Stephanie Pfänder, Michael Adamzik, Björn Koos, Dominik Ziehe, Maha Bazzi, Lars Bergmann, Alexander von Busch, Stefan F. Ehrentraut, Martin Eisennacher, Sai Spoorti Ramesh, Helge Haberl, Melanie Meersch-Dini, Katrin Marcus, Katharina Rump, Jens-Christian Schewe, Jennifer Orlowski, Britta Marco, Matthias Unterberg, Daniel Kühn, Alexander Wolf, Birgit Zuelch

**Affiliations:** 1 Klinik für Anästhesiologie, Intensivmedizin und Schmerztherapie, Universitätsklinikum Knappschaftskrankenhaus Bochum, In der Schornau 23-25, 44892 Bochum, Germany; 2 Zentrum für Künstliche Intelligenz, Medizininformatik und Datenwissenschaften, Universitätsklinikum Knappschaftskrankenhaus Bochum, Bochum, Germany; 3 Klinik für Anästhesiologie und operative Intensivmedizin, Universitätsklinikum Bonn, Bonn, Germany; 4 Klinik für Anästhesiologie, operative Intensivmedizin und Schmerztherapie, Universitätsklinikum Münster, Münster, Germany; 5 Centrum für Translationale Medizin, Medizinische Klinik I, Marien Hospital Herne, Universitätsklinikum der Ruhr-Universität Bochum, Herne, Germany; 6 Medizinisches Proteom-Center, Ruhr Universität Bochum, Medizinische Fakultät, Bochum, Germany; 7 Center for Protein Diagnostics (PRODI), Medical Proteome Analysis, Ruhr Universität Bochum, Bochum, Germany; 8 Research Unit Emerging Viruses, Leibniz Institute of Virology (LIV), Hamburg, Germany; 9 University of Lübeck, Lübeck, Germany

**Keywords:** COVID-19, Sepsis, RAF, MEK, ERK, MAPK, PLA

## Abstract

**Purpose:**

There is evidence that lower activity of the RAF/MEK/ERK network is associated with positive outcomes in mild and moderate courses of COVID-19. The effect of this cascade in COVID-19 sepsis is still undetermined. Therefore, we tested the hypothesis that activity of the RAF/MEK/ERK network in COVID-19-induced sepsis is associated with an impact on 30-day survival.

**Methods:**

We used biomaterial from 81 prospectively recruited patients from the multicentric CovidDataNet.NRW-study cohort (German clinical trial registry: DRKS00026184) with their collected medical history, vital signs, laboratory parameters, microbiological findings and patient outcome. ERK activity was measured by evaluating ERK phosphorylation using a Proximity Ligation Assay.

**Results:**

An increased ERK activity at 4 days after diagnosis of COVID-19-induced sepsis was associated with a more than threefold increased chance of survival in an adjusted Cox regression model. ERK activity was independent of other confounders such as Charlson Comorbidity Index or SOFA score (HR 0.28, 95% CI 0.10–0.84, p = 0.02).

**Conclusion:**

High activity of the RAF/MEK/ERK network during the course of COVID-19 sepsis is a protective factor and may indicate recovery of the immune system. Further studies are needed to confirm these results.

**Supplementary Information:**

The online version contains supplementary material available at 10.1007/s15010-024-02325-7.

## Introduction

With the onset of the COVID-19 pandemic, a new disease pattern emerged [1]. While the majority of patients had asymptomatic to mild illness [2, 3], some patients experienced severe progressions associated with high mortality [4, 5]. The development of critical illness was characterized by an onset of acute respiratory distress syndrome (ARDS) and multiorgan failure [6] that fulfilled the criteria for sepsis [7]. Why some patients develop critical illness and succumb to COVID-19 induced sepsis and others present only mild symptoms is still not well understood and various signaling networks have been investigated to this end [8, 9]. A well-studied and frequently utilized approach by other viruses is the RAF/MEK/ERK network (rapidly accelerated fibrosarcoma/mitogen-activated protein kinase/extracellular signal-regulated kinase network) [10–13]. For COVID-19, exploitation of this specific pathway by the SARS-CoV-2 virus has also been identified for the early phase of infection, leading to increased viral replication [14]. Further studies showed that MEK inhibitors, such as those being tested against the influenza virus [15], can also alleviate viral replication of SARS-CoV-2 and subsequent cytokine release, which is considered to be a major driver of critical disease progression [16–18]. To date, it is unclear whether the suggested protective effects of a suppressed RAF/MEK/ERK activity are also applicable to the later phase of COVID-19-induced sepsis in humans [19, 20]. With the persistently poor prognosis of critically ill COVID-19 patients, this would provide an opportunity for a potential therapeutic approach. Therefore, we tested the hypothesis that activity of the RAF/MEK/ERK network in COVID-19-induced sepsis is associated with an impact on 30-day survival.

## Methods

### Study design

The multicentric CovidDataNet.NRW study (German Clinical Trial Registry No. DRKS00026184) prospectively enrolled patients with sepsis because of a COVID-19 infection, meeting the sepsis-3 criteria. These patients were recruited from the Intensive Care Units (ICUs) of three distinct university hospitals situated in the German state of North Rhine-Westphalia. Ethical approval for this study was granted by the Ethics Committee of the Medical Faculty of Ruhr-University Bochum (Registration No. 18–6606–BR) or the relevant ethics committee at each respective study center. Patients were recruited between March 1, 2020 and October 30, 2022.

This study encompassed adult patients diagnosed with COVID-19-induced sepsis within the preceding 36 h, in accordance with the current sepsis-3 definition, which requires a suspected or proven infection along with a Sequential Organ Failure Assessment (SOFA) score increase of at least two points. The patient cohort comprised a mix of surgical and medical cases admitted to the ICU. Exclusion criteria included: (1) age less than 18 years at the time of ICU admission, (2) withdrawal or withholding of consent, and (3) discontinuation of treatment. Patients with an unknown 30-day survival status were excluded from subsequent analysis.

### Clinical data and patient characteristics

Electronic medical data, including vital signs, laboratory results, point-of-care diagnostics, demographic information, and the duration of ICU or hospital stays, were meticulously captured within a comprehensive database using CentraXX software, developed by Kairos GmbH in Bochum, Germany. This data collection process adhered to the pseudonymization procedures mandated by the ethics committee.

In cases of missing data, a qualified physician at each respective clinic conducted individual patient record investigations. Where deemed appropriate, data from ± 12 h of sepsis onset were included to ensure completeness. The SOFA scores were manually calculated by experienced physicians at each recruitment site. SARS-CoV-2 PCR Test were performed via nasopharyngeal swaps by the attending physicians and cycling time values (CT value) were reported. The number of cycles was limited to 40. If no viral RNA was detected by that time, the patient was considered negative. Throughout their ICU stay, each patient underwent comprehensive microbiological monitoring, which included surface swabs, tracheal secretions, urine cultures, and bronchial secretions or bronchoalveolar lavage where feasible. If a pathogen was detected and deemed in need of treatment by the attending physician (i.e., with no evidence of contamination or colonization), the patient was classified as superinfected. The findings were stratified according to the day of sample collection (during the overall ICU stay versus after day 4).

### Extraction of peripheral blood mononuclear cells (PBMCs)

Blood was drawn from the patients at study inclusion and after 4, 8 and 14 days. Peripheral blood mononuclear cell (PBMCs) were isolated from EDTA stabilized blood samples using Ficoll density gradient centrifugation (GE Healthcare Europe, Freiburg, Germany). The phase containing PBMCs was collected and washed with PBS. Subsequent to erythrocyte lysis, and PBS (phosphate buffered saline) washing, the PBMCs were stored at – 196 °C until use. Upon thawing, cells were counted, spun onto microscopic slides using a cytospin (Cellspin II, Tharmac, Wiesbaden, Germany), and then fixed using 4% formaldehyde solution.

### Proximity ligation assay for pERK

The Proximity Ligation Assay (PLA) to assess the phosphorylation level of ERK was performed as described previously [21]. Briefly, PBMCs were permeabilized using 1% Triton-X in PBS. Partial unfolding of the target proteins was done by incubating the slides with 1% SDS in PBS. After subsequent washing slides were blocked using the Duolink Block (Sigma). Primary antibodies against ERK (1:100, #4696, Cell Signaling Technology, Danvers, MA) and pERK (1:100, #4370, Cell Signaling Technology) were incubated at 4 °C over night. After another round of washing, we incubated the slides with secondary proximity probes (anti-mouse and anti-rabbit, NaveniFlex 100 MR, Navinci Diagnostics, Uppsala, Sweden) for 1h at 37 °C. The Unfold PLA reaction was conducted as per manufacturers recommendations (Navinci Diagnostics). After mounting the slides with slow fade antifade reagent (S36940, Thermo Fisher) and counterstaining the nuclei with DAPI we evaluated the pERK level using a IX51 Microscope (Zeiss, Germany).

### Image analysis

Analysis of the images was undertaken using FIJI and the Cell Profiler software. First, maximal intensity projections were performed and channels were merged using FIJI. The images were then imported to Cell Profiler where the modules primary object identification, secondary object identification and relate object modules were used to quantify the PLA signals per cell. As we did no cytoplasmic counterstain, we estimated a cell to be of 30 pixel diameter around the nucleus. Only slides with at least 50 cells were evaluated.

### Cytokine measurements

The following cytokines were measured on day 1 and day 4 by a customized human LegendPlex assay (BioLegend, San Dieago, CA): Inteleukin-1 beta (IL-1b), Interleukin-6 (IL-6), Interleukin-10 (IL-10), Interleukin-18 (IL-18), Interferon gamma (INF-γ), tumor necrosis factor alpha (TNF-α), Interferon alpha2 (INF-α2).

### Plasma proteomics

The liquid chromatography-tandem mass spectrometry (LC–MS/MS) analyses were conducted as described before [22]. Briefly, plasma samples were prepared according to the SP3 protocol [23] and analyzed using an Ultimate 3000 RSLCnano HPLC coupled to an Orbitrap Exploris 240 mass spectrometer (both Thermo Scientific). The peptides were separated using a 37 min gradient from 4 to 28% acetonitrile in 0.1% formic acid and were measured using data independent acquisition. DIA-NN (v.1.8) was used for protein quantification with an in-house created spectral-library generated from plasma DDA measurements with FragPipe (v.17.1).

### Statistics

Continuous variables are presented as means ± standard deviation in the case of normal distribution and as median and interquartile range (25th; 75th percentile) in the case of non-normally distributed variables. The statistical analyses were performed using the software R (R version 3.5.3; The R Foundation for Statistical Computing; http://www.R-project.org). A two-sided p-value < 0.05 was considered statistically significant. Confidence intervals (CIs) were calculated with 95% coverage.

## Results

### Cohort description

**Fig. 1:**
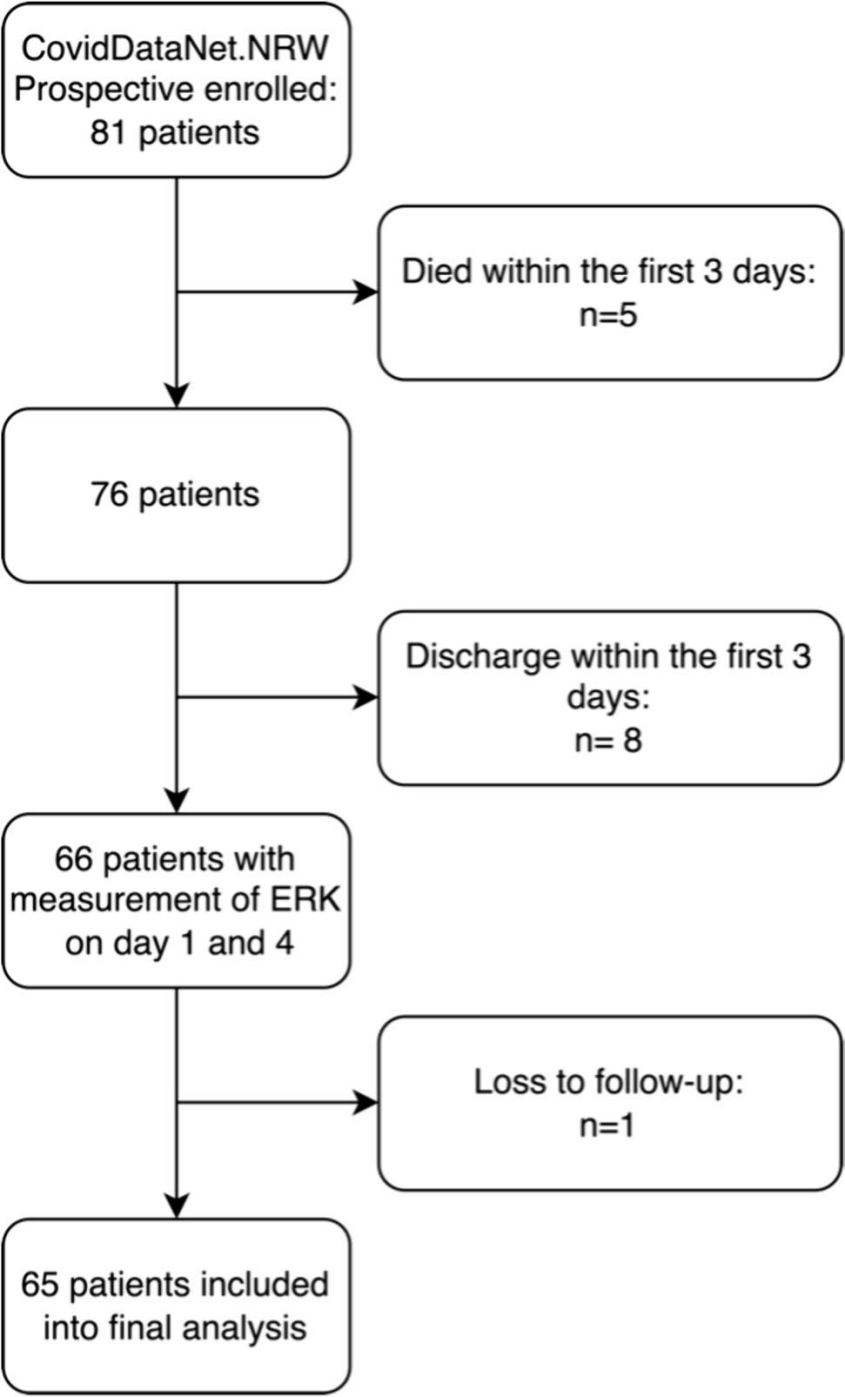
81 patients with confirmed Covid-19 induced sepsis in the intensive care unit were prospectively enrolled. 3 patients died within the first 3 days, 8 patients were discharged and one patient could not be followed up. 65 patients were included in the final analysis of ERK activity on day 4

81 SARS-CoV-2 positive septic patients from three ICUs were included in our study (Fig. 1). The cohort consisted of 34 male patients (42%) and mean age was 58 (± 15) years. The median SOFA score at study inclusion was 9.5 (IQR: 5–12) and the Charlson Comorbidity Index (CCI) was 3 (IQR: 2–4). The 30d mortality rate of the cohort was 38%. For further base characteristics see Table 1.

**Table 1:** Base characteristics of the Covid-19 sepsis cohort (n = 81)

Age *years* (IQR)	58.0 (49–79)
Male sex, n (%)	34 (42%)
Admission SOFA Score (IQR)	9 (5–12)
ICU length of stay, *days* (IQR)	20 (10–31)
Charlson Comorbidity Index (IQR)	3.0 (2.0–4.0)
Comorbid condition, n (%)	
Hypertension	45 (56%)
Chronic kidney disease	11 (14%)
COPD	4 (5%)
Other lung disease	13 (16%)
Diabetes mellitus	25 (31%)
Obesity	35 (43%)
Cardiovascular disease	18 (22%)
Malignancy	7 (9%)
Organ transplantation	7 (9%)
Laboratory value, day 1	
Hemoblobin, mg/dl (IQR)	11.4 (9.2–12.5)
Thrombocytes, 10^3^/µl (IQR)	213.5 (135.1–275.1)
Quick, % (IQR)	80.5 (75.5–89.3)
C-reactive protein, mg/dl (IQR)	12.6(7.3–20.2)
Procalcitonin, ng/ml (IQR)	0.33(0.14–0.85)
Lactate, mmol/l (IQR)	1.54(1.31–1.93)
White blood cells, n/µl (IQR)	9.70(5.85–15.25)
30-day mortality, n (%)	25 (38%)

Data are presented as n (%); mean (± SD); median (IQR (25th, 75th percentile))

### Impact of ERK activity on 30-day survival

**Fig. 2:**
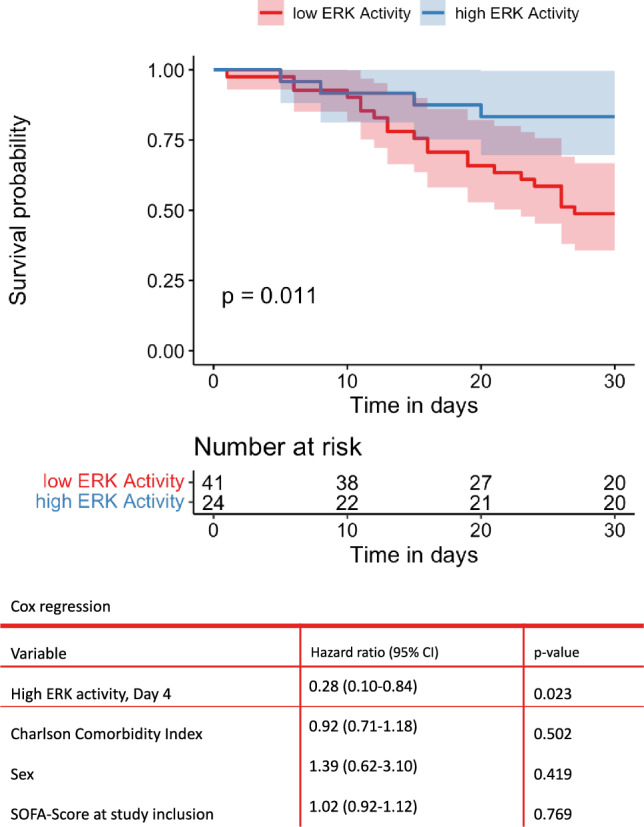
The Kaplan–Meier curve of 30-day survival as a function of ERK activity is plotted. Patients with increased ERK activity have a significantly lower mortality rate (p = 0.011). In the following Cox regression, the ERK activity was adjusted for CCI, gender and the SOFA score at the time of inclusion. Increased ERK activity was identified as the only significant, protective factor

Looking at the Kaplan–Meier curve (Fig. 2), a significant difference in survival depending on the ERK activity on day 4 after sepsis diagnosis was observed. While only 20 of 41 patients with reduced pERK on day 4 were alive after 30 days (30d survival = 48.78%), 20 of 24 patients with increased pERK survived for 30 days (30d survival = 83.33%), which was statistically significant (p = 0.011). Regarding the rate of superinfections, there was no significant difference between groups (p = 0.17).

The Cox regression analysis included the factors CCI, sex, and SOFA score at sepsis onset in addition to ERK activity at day 4 of sepsis (Fig. 2). Here, ERK activity was found to be a protective factor with respect to 30-day survival with a hazard ratio of 0.28 (95% CI 0.10–0.84) and remained the only statistically significant factor (p = 0.02).

**Fig. 3:**
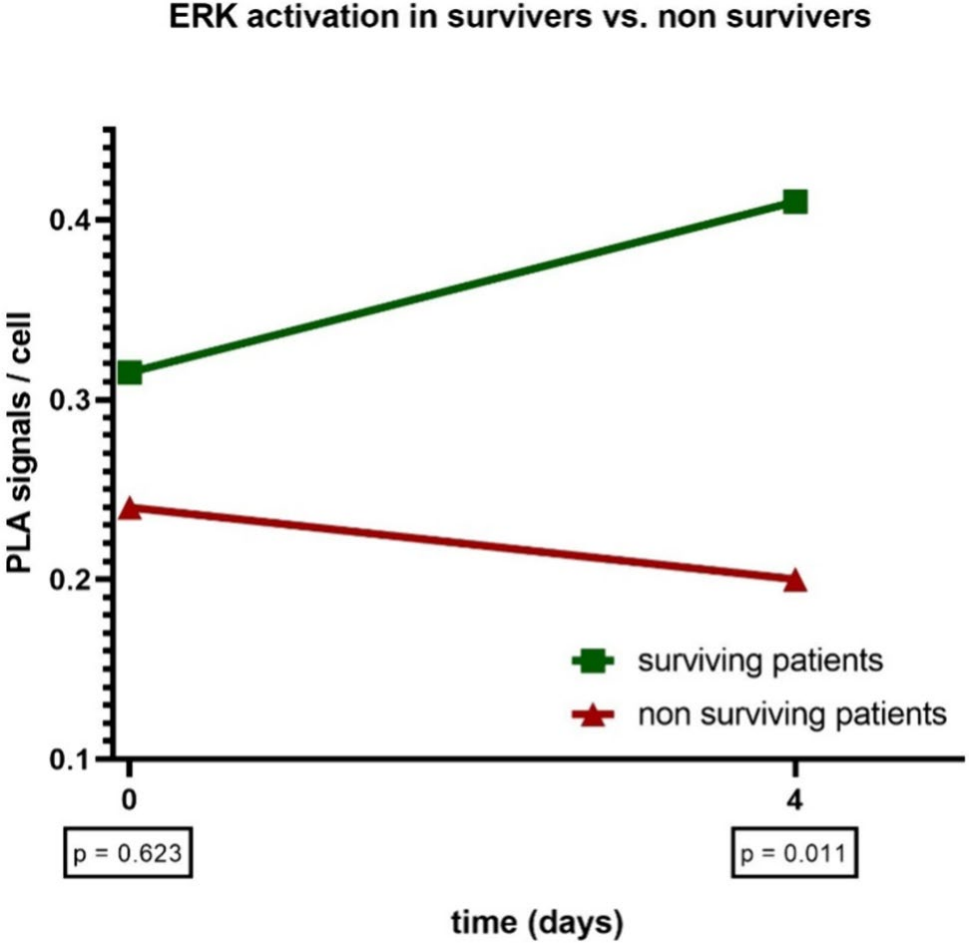
The trajectories of ERK activity in the survivors and non-survivors are presented. While there is no significant difference between the two groups on day 1 of sepsis, significantly higher ERK activity is observed in the survivor group on day 4 (p = 0.011)

Regarding the trend of ERK activity in relation to survival, we observed that ERK activity decreases on average in the "non-survivors" while it remained almost constant in the "survivors". This resulted in a significant difference in ERK activity on day 4 between the survivors and the deceased (p = 0.011) (Fig. 3).

### Classification of the cohort according to ERK activity

When the cohort was classified according to their ERK activity at day 4, no significant group differences except for mortality rate were observed. However, a trend towards a higher concentration of the pro-inflammatory cytokine IL-6 in association with high ERK activity was seen (p = 0.05). In contrast, patients with high ERK activity showed a decrease in CRP (p = 0.092). Of note, the SARS-CoV-2 PCR test CT value increased significantly from day 1 to day 4 in both cohorts. While the CT value increased from 26.13 to 28.87 (p = 0.018) within patients with high ERK activity, an increase from 24.17 to 28.79 (p < 0.001) in patients with low ERK was evident. However, there was no significant difference considering the mean increase from day 1 to day 4 between both groups (4.62 vs. 2.73, p = 0.23) Table 2.

**Table 2:** Classification of the cohort according to their ERK activity on day 4

Variable	High ERK activity, n = 24	Low ERK activity, n = 41	p-value
Age *years.* (IQR)	55.5 (45.25–59.25)	63 (48–74)	0.5714
Male sex, n (%)	8 (33%)	19 (46%)	0.1493
Admission SOFA Score (IQR)	9 (5.75–12.00)	10 (7–12)	0.3934
ICU length of stay, *Days* (IQR)	23.5 (7.5–39.25)	19 (11–29)	0.6584
Charlson Comorbidity Index (IQR)	3.0 (2.0–3.5)	3.0 (2.0–4.0)	0.6812
SARS-CoV-2 PCR Test, CT-value			
Day 1	26.13 (± 6.3)	24.17 (± 5.33)	0.3138
Day 4	28.87 (± 6.4)	28.79 (± 6.42)	0.9715
Bacterial superinfection, n (%)			
Overall ICU stay	5 (21%)	16 (39%)	0.1731
≥ Day 4	3 (13%)	7 (17%)	0.7331
Comorbid condition, n (%)			
Hypertension	10 (42%)	24 (58%)	0.2095
Chronic kidney disease	2 (8%)	6 (15%)	0.6995
Chronic obstructive pulmonary disease (COPD)	3 (13%)	1 (2%)	0.1383
Other lung disease	2 (8%)	6 (15%)	0.6995
Diabetes mellitus	8 (33%)	13 (32%)	0.9999
Obesity	15 (63%)	17 (42%)	0.1271
Cardiovascular disease	2 (8%)	11 (27%)	0.1088
Malignancy	2 (8%)	3 (7%)	0.9999
Organ transplantation	2 (8%)	3 (7%)	0.9999
Laboratory values			
C-reactive protein, mg/dl (IQR)			
Day 1	13.12 (6.65–23.03)	12.37 (7.34–15.40)	0.8044
Day 4	4.02 (3.11–14.21)	9.51 (4.82–20.26)	0.0923
Procalcitonin, ng/ml (IQR)			
Day 1	0.22 (0.14–0.64)	0.33 (0.15–1.70)	0.3927
Day 4	0.25 (0.11–0.41)	0.31 (0.13–1.43)	0.2088
Lactate, mmol/l (IQ R)			
Day 1	1.49 (1.32–1.74)	1.58 (1.31–1.94)	0.4881
Day 4	1.43 (1.34–1.77)	1.73 (1.29–2.15)	0.5285
White blood cells, n/µl (IQR)			
Day 1	9.40 (6.15–12.15)	10.10 (5.90–17.70)	0.5038
Day 4	11.55 (9.60–14.40)	13.92 (9.70–17.02)	0.5013
Cytokine values			
IL-1b, pg/ml (IQR)			
Day 1	1.41 (0.21–137.22)	1.45 (0.36–10.98)	0.9918
Day 4	0.34 (0.00–62.02)	2.18 (0.00–6.23)	0.6711
IL-6, pg/ml (IQR)			
Day 1	182.4 (10.4–2854.5)	41.1 (14.25–914.4)	0.5048
Day 4	1024.0 (95.85–9843.4)	100.5 (15.96–928.3)	0.0504
IL-10, pg/ml (IQR)			
Day 1	4.58 (2.89–112.17)	5.57 (1.38–20.66)	0.6223
Day 4	19.57 (5.02–45.47)	6.90 (3.73–17.75)	0.3739
IL-18, pg/ml (IQR)			
Day 1	152.5 (46.9–291.9)	116.0 (5.2–362.7)	0.696
Day 4	290.5 (183.2–711.5)	279.6 (73.9–780.8)	0.7669
INF-γ, pg/ml (IQR)			
Day 1	15.21 (7.79–195.43)	7.63 (3.05–66.91)	0.2461
Day 4	11.97 (8.32–86.34)	13.51 (4.25–19.19)	0.6523
TNF-α, pg/ml (IQR)			
Day 1	3.60 (1.80–106.53)	2.43 (1.27–5.74)	0.2221
Day 4	2.94 (1.82–27.95)	3.65 (2.11–7.48)	0.6782
INF-α2, pg/ml (IQR)			
Day 1	4.09 (1.45–8.67)	1.87(1.22–7-67)	0.3718
Day 4	4.02 (1.61–12.79)	3.33 (2.06–5.63)	0.4992
30-Day mortality, n (%)	4 (17%)	21 (51%)	**0.0079**

Significant p-value is marked in bold

Regarding plasma proteomics, no significant differences in abundant proteins was observed between patients with high and low ERK activity at day 1 or day 4 (Supplementary Fig. 1).

## Discussion

The main finding of this study is: Increased ERK activity on day 4 of COVID-19 induced sepsis is associated with improved 30-day survival.

Despite extensive research, the precise pathophysiological mechanisms of (viral) sepsis remain incompletely elucidated. While pro- and anti-inflammatory cascades are simultaneously activated at the beginning of sepsis, hyperinflammation predominates during the early stages of sepsis, leading to a cytokine storm, vasodilation, oxygen deprivation and organ dysfunction [7]. In the later phase, sepsis can result in sepsis-associated immunosuppression or even immunoparalysis [24].

The RAF/MEK/ERK (also known as MAPK) network plays a crucial role in the inflammatory response as demonstrated by correlations with a range of cytokines [25]. The MAPK network is extensively studied in mammalian cells and also plays a critical role in various cellular functions besides the immune response such as cell proliferation or apoptosis. Hence, this network is often used as a proxy for the activation state of these cellular functions and the activation of ERK (or MAPK) is mostly used to evaluate the activation of this network.

Based on this, ERK activation on day 4 could be viewed as a restarting of the MAPK signaling, which in turn could be indicating a status of regaining immune balance of the patients. This, by preventing immune paralysis, could lead to a higher survival, hence explaining our results. The correlation between cytokines and ERK activation that we observed supports this hypothesis (see Supplementary Table 1).

However, an alternative perspective should be considered. Several articles have described the utilization of the RAF/MEK/ERK network by various viruses, including influenza [12, 26–28]. In case of the SARS-CoV-2 virus, MAPK signaling facilitates virus entry into the cell and promotes virus replication [16]. Therefore, the RAF/MEK/ERK network has been discussed as a valuable target in COVID-19 therapy [16–18]. Since MEK exclusively targets ERK, it serves as a significant pharmacological target for inhibiting the MAPK [25] network and many inhibitors of MEK have been developed [29]. Consequently, several authors have tested the hypothesis that applying MEK-inhibitors in COVID-19 infected cells could reduce viral replication and hyperinflammation [14, 16]. The authors showed that treatment with MEK antagonists inhibited inflammatory cytokines in vitro and an improved clinical severity score in vivo. These reports seem to contradict our main finding, namely that activation of ERK is beneficial for patients with severe COVID-19.

In order to discuss this, it is worth emphasizing that the previous studies on RAF/MEK/ERK were restricted to patients with moderate-to-severe COVID-19, i.e., without viral sepsis, and those receiving intensive care treatment or needing ventilator support were excluded. In these stages of COVID-19 it may be beneficial to block viral entry into the host cells and also curb a hyperinflammatory response in order to contribute to regaining immune balance of the patient, thus preventing a viral sepsis. During later stages of the disease, i.e. COVID-19 induced sepsis, activation of ERK, and hence increased pro-inflammation, may be beneficial in order to regain immune balance from the immunosuppressive state. In our work, high as well as low ERK activity patients showed an increase of the CT value. This indicates that there has already been a decrease in viral load in both cohorts. While effective antiviral therapy before and after the onset of viral sepsis is essential, modulation of the dysregulated immune response in COVID-19 sepsis is emerging as an area of major importance in improving patient outcomes [30] and may be more important for the survival of the patient at this stage. Therefore, while inhibiting ERK seems to be a promising therapeutic strategy in patients with moderate COVID-19, the same therapeutic strategy may be harmful for septic COVID-19 patients.

It is noteworthy that, upon stratifying the cohort based on their ERK activity in relation to the plasma proteome, no significant differences were observed. Despite detailed proteomic studies revealing characteristic changes in COVID-19 [31], we were unable to attribute these changes to the activity of the MAPK network in the late phase of infection. This lack of association may be explained by substantial interindividual heterogeneity in the proteome, compounded by the inherent critical condition of our cohort. While previous literature has underscored the value of proteomic analysis in categorizing COVID-19 courses into asymptomatic, mild, and severe [32], such categorization proved inherently unfeasible in our study, given that COVID-19 sepsis consistently accompanies a severe course.

As far as we know, the present study is the first to describe the impact of RAF/MEK/ERK network on outcome focusing on patients with COVID-19 sepsis. In summary, our findings contribute to the understanding of both the complexity and time sensitivity of the immune response in COVID-19 sepsis. Further research is required to obtain more nuanced perspectives and refine understanding COVID-19 sepsis and possible therapeutic targets.

## Limitations

Although our study benefits from prospective enrollment and high-quality data, it is constrained by a relatively small sample size. Furthermore, the homogeneity of our cohort and the pronounced initial disease severity limit the generalizability of our findings. As a result, some established COVID-19 risk factors did not reach statistical significance in our analysis. Therefore, it is imperative to validate our results in a larger and more diverse population. This also applies to the rate of superinfections during COVID-19 sepsis. A strength of our study is the availability of comprehensive microbiological monitoring for the patients. The observed superinfection prevalence of 31% (21 out of 65 patients) aligns with findings reported in the literature [33]. We did not detect any significant differences between groups concerning MAPK network activity and superinfection rates, although there were hints for a more frequent occurrence in the cohort with lower ERK activity (high ERK activity 21% vs. low ERK activity 39%, p = 0.17). However, due to the small sample size, these results, as well, must be interpreted with caution and require validation in a larger cohort. In addition, it should be noted that the trajectory of ERK activity presented here represents only two snapshots. Both the activity at the onset of the disease and in the later stages are of interest to achieve a comprehensive understanding of the impact of the MAPK network throughout the course of the illness.

## Conclusion

High activity of the MAPK network in the late phase of COVID-19 sepsis seems to be beneficial to the patient. Reestablishing balance of the immune system is a potential interpretation. Due to the variety of functions of ERK, further studies are needed to confirm these results.

## Supplementary Information

Below is the link to the electronic supplementary material.

Supplementary file 1 (DOCX 186 KB)

Supplementary file 2 (DOCX 13 KB)

## Data Availability

The dataset analyzed during the current study is available from the corresponding author on reasonable request.

## Abbreviations

CCI: Charlson Comorbidity Index
CI: Confidence interval
COVID-19: Coronora virus disease 2019
COPD: Chronic obstructive pulmonary disease
CRP: C-reactive protein
ERK: Extracellular signal-regulated kinase
ICU: Intensive Care Unit
IL-1b: Inteleukin-1 beta
IL-6: Interleukin-6
IL-10: Interleukin-10
IL-18: Interleukin-18
INF- α2: Interferon alpha2
INF- γ: Interferon gamma
IQR: Interquatile range
PBMC: Peripheral blood mononuclear cells
PBS: Phosphate buffered saline
pERK: Phosphorylated extracellular signal-regulated kinase
PLA: Proximity Ligation Assay
MAPK: Mitogen-activated protein kinase
MEK: Mitogen-activated protein kinase
RAF/MEK/ERK: Rapidly accelerated fibrosarcoma/mitogen-activated protein kinase/extracellular signal-regulated kinase
SARS-CoV-2: Severe acute respiratory syndrome coronavirus type 2
SOFA: Sequential Organ Failure Assessment
TNF-α: Tumor necrosis factor alpha
