# Supplementary material for: Activation of the MAPK network provides a survival advantage during the course of COVID-19-induced sepsis: a real-world evidence analysis of a multicenter COVID-19 Sepsis Cohort
Source: Infection. 2024 Jun 19;53(1):107–15. doi: 10.1007/s15010-024-02325-7 (PMC11825614; doi:10.1007/s15010-024-02325-7)
Supplement: Supplementary file 1 — (DOCX 186 KB) [file 15010_2024_2325_MOESM1_ESM.docx]

**Supp.-Figure 1:**


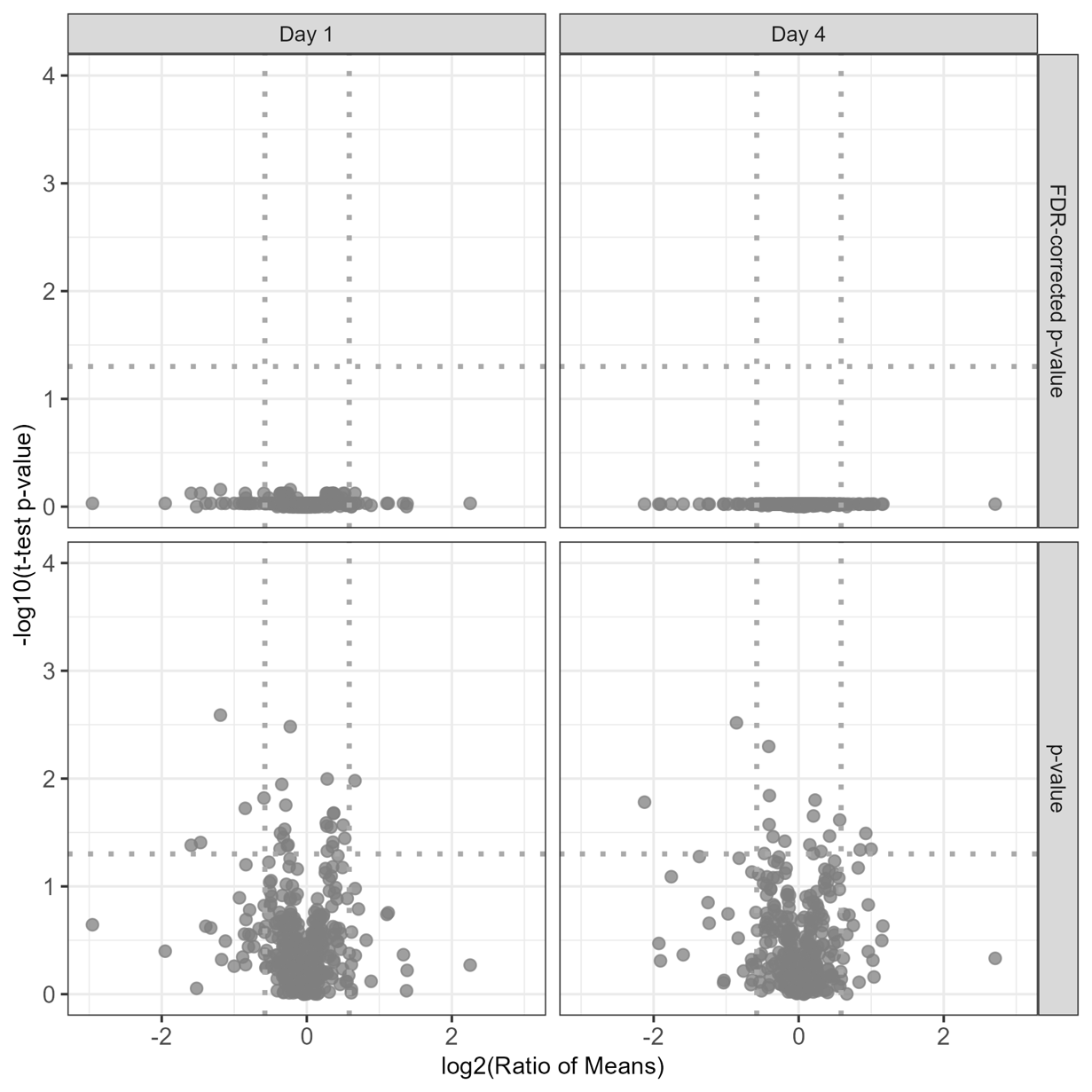


Volcano plots representing the results of t-test analysis (high vs. low ERK activity, analyzed separately for days 1 and 4). No significantly differentially abundant proteins were detected according to filter criteria p_FDR_-value ≤ 0.05 (corrected according to Benjamini–Hochberg) and an absolute ratio of means ≥ 1.5.
