# Supplementary material for: Activation of the MAPK network provides a survival advantage during the course of COVID-19-induced sepsis: a real-world evidence analysis of a multicenter COVID-19 Sepsis Cohort
Source: Infection. 2024 Jun 19;53(1):107–15. doi: 10.1007/s15010-024-02325-7 (PMC11825614; doi:10.1007/s15010-024-02325-7)
Supplement: Supplementary file 2 — (DOCX 13 KB) [file 15010_2024_2325_MOESM2_ESM.docx]

**Supp.-Table 1: Pearson correlation analysis of ERK activity and cytokine levels, n=65**

| **Variable** | ERK activity, day 1 |
| --- | --- |
| **Cytokine values, day 1** |  |
| IL-1b | 0.61 (0.39-0.76, p<0.001) |
| IL-6 | 0.58 (0.35-0.74, p<0.001) |
| IL-10 | 0.63 (0.43-0.78, p<0.001) |
| IL-18 | -0.05 (-0.34-0.24, p=0.722) |
| INF- γ | 0.69 (0.51-0.81, p<0.001) |
| TNF-α | 0.79 (0.78-0.87, p<0.001) |
| INF- α2 | 0.23 (-0.06-0.49, p=0.121) |
